# Molecular and Clinicopathological Profiling of Clear Cell Renal Cell Carcinoma with Rhabdoid Features: An Integrative Pathway-Based Stratification Approach

**DOI:** 10.3390/cancers17172744

**Published:** 2025-08-23

**Authors:** Zhichun Lu, Qing Zhao, Huihong Xu, Mark H. Katz, David S. Wang, Christopher D. Andry, Shi Yang

**Affiliations:** 1 Department of Pathology & Laboratory Medicine, Boston Medical Center, Boston University Chobanian & Avedisian School of Medicine, Boston, MA 02108, USA; grace.zhao@bmc.org (Q.Z.); chris.andry@bmc.org (C.D.A.); shi.yang@bmc.org (S.Y.); 2 Department of Pathology & Laboratory Medicine, Boston VA Health Care System, Boston, MA 02130, USA; 3 Department of Urology, Boston Medical Center, Boston University Chobanian & Avedisian School of Medicine, Boston, MA 02108, USA; mark.katz@bmc.org (M.H.K.); david.wang@bmc.org (D.S.W.)

**Keywords:** clear cell renal cell carcinoma, rhabdoid features, pathway-based molecular stratification, DNA damage repair, chromatin remodeling, PI3K/AKT/mTOR, MAPK pathway, *BAP1*, *SETD2*, *PTEN*, *FGFR*, *MAP2K2*, *NOTCH*

## Abstract

**Background**: Clear cell renal cell carcinoma with rhabdoid features (ccRCC-R) is a highly aggressive variant of renal cell carcinoma that carries a poor prognosis and limited treatment options. **Methods**: To better define the clinicopathologic and molecular landscape of ccRCC-R, we conducted an integrated clinicopathologic and molecular study of 17 tumors of ccRCC-R, utilizing comprehensive histomorphologic evaluation, immunohistochemistry, and targeted next-generation sequencing (NGS). **Results**: Histologically, all tumors demonstrated classic clear cell renal cell carcinoma morphology with focal to extensive rhabdoid differentiation, characterized by eccentrically located nuclei, prominent nucleoli, abundant eosinophilic cytoplasm, and paranuclear intracytoplasmic inclusion. Architectural alterations, including solid/sheet-like, alveolar/trabecular, and pseudopapillary growth patterns, were frequently observed. Immunohistochemically, tumors commonly exhibited loss of PAX8 and Claudin4 expression, preserved cytokeratin AE1/AE3 staining, and diffuse membranous CAIX expression. Frequent loss of SMARCA2 with retained SMARCA4 supported aberrations in chromatin remodeling. Unsupervised hierarchical clustering based on pathway-specific somatic mutations identified four distinct molecular subgroups defined by recurrent alterations in (1) DNA damage repair (DDR) genes, (2) chromatin remodeling genes, (3) PI3K/AKT/mTOR signaling components, and (4) MAPK pathway genes. Clinicopathologic correlation revealed that each subgroup was associated with unique biological characteristics and suggested distinct therapeutic vulnerabilities. **Conclusions**: Our findings underscore the molecular heterogeneity of ccRCC-R and support the utility of pathway-based stratification for guiding precision oncology approaches and biomarker-informed clinical trial design.

## Simple Summary

Clear cell renal cell carcinoma with rhabdoid features (ccRCC-R) is a rare and aggressive cancer with limited treatment options. In this study, 17 cases were analyzed using a detailed histomorphologic review, immunohistochemistry, and targeted next-generation sequencing. The tumors were grouped into four distinct molecular subtypes based on common pathway-specific mutations involving DNA damage repair, chromatin remodeling, and PI3K/AKT/mTOR and MAPK pathways. Each group showed unique clinical and biological features, highlighting potential targets for personalized therapy. This work emphasizes the importance of molecular profiling in understanding ccRCC-R and developing future precision treatment strategies.

## 1. Introduction

Clear cell renal cell carcinoma with rhabdoid features (ccRCC-R) represents a histologically and biologically aggressive variant of renal cell carcinoma, associated with poor clinical outcomes and limited therapeutic options [1,2,3]. Although rhabdoid differentiation is observed in approximately 4–5% of ccRCC tumors, it is an established independent adverse prognostic factor, correlating with higher tumor stage, increased nuclear grade in the non-rhabdoid component, and elevated cancer-specific mortality [1,4].

Despite its clinical relevance, the underlying immunophenotypic and molecular mechanisms driving rhabdoid differentiation in ccRCC remain poorly understood, in part due to pronounced phenotypic variability and significant intratumoral heterogeneity [5,6]. Prior genomic studies have reported recurrent alterations in key molecular pathways in ccRCC-R, including chromatin remodeling genes, DNA damage repair (DDR) pathways, and components of oncogenic signaling cascades such as PI3K/AKT/mTOR and MAPK [7,8]. However, a systematic pathway-based approach to molecular classification and therapeutic stratification has not been thoroughly explored.

Given the aggressive clinical behavior and relative resistance of ccRCC-R to conventional therapies, there is an urgent need for precision oncology strategies tailored to its unique biology [3,9,10]. In this study, we conducted an integrated histomorphologic, immunophenotypic, and targeted next-generation sequencing (NGS) analysis of ccRCC-R. Through hierarchical clustering based on pathway-specific somatic alterations, we identified distinct molecular subtypes that may inform biomarker-driven clinical decision-making and targeted therapy selection.

## 2. Materials and Methods

### 2.1. Tumor Selection and Study Design

This study was approved by the Institutional Review Board of Boston University Medical Center. A retrospective analysis was performed on nephrectomy specimens diagnosed as clear cell renal cell carcinoma (ccRCC) and surgically resected between January 2007 and July 2022. The inclusion criteria were as follows: (1) tumor size > 4 cm and (2) high-grade nuclear features, defined as WHO/ISUP grade 3 or 4 or Furman grade 3 or 4.

Tumors of ccRCC with rhabdoid features (ccRCC-R) were identified based on the presence of a rhabdoid component comprising an equivalent of more than 5% of the total tumor volume, in conjunction with a recognizable low-grade ccRCC component. One representative formalin-fixed paraffin-embedded (FFPE) tumor block per tumor was selected for histologic review, immunohistochemical analysis, and targeted next-generation sequencing (NGS).

Immunohistochemistry and molecular profiling were conducted using a defined panel and the Oncomine™ Comprehensive Assay. Relevant clinicopathological information, including patient age, sex, laterality of the tumor, type of surgery, adjuvant and systemic therapy, time of the follow-up, and clinical outcomes, was retrospectively collected from electronic medical records.

### 2.2. Histomorphologic Evaluation

Tumor size was recorded based on gross examination of the resected specimens. Comprehensive histopathologic assessment was performed on hematoxylin and eosin (H&E)-stained sections. Nuclear grading was assigned according to the World Health Organization (WHO)/International Society of Urological Pathology (ISUP) grading system. Additional histologic parameters included predominant architectural patterns of the rhabdoid component, the extent of rhabdoid and sarcomatoid differentiation, the presence of tumor necrosis, and high-grade cytologic features [11].

Other pathologic features were systematically evaluated, including renal sinus invasion, perirenal adipose tissue invasion, adrenal gland involvement, vascular and renal vein invasion, regional lymph node metastasis, and surgical margin status. Tumors were staged according to the American Joint Committee on Cancer (AJCC) 8th Edition TNM classification [12].

### 2.3. Immunohistochemistry

Immunohistochemical analysis was performed on formalin-fixed paraffin-embedded (FFPE) tissue sections using automated staining platforms (Ventana Medical Systems, Tucson, AZ) with prediluted antibodies. The antibody panel included cytokeratin (AE1/AE3, PCK26), PAX8 (MRQ-50), carbonic anhydrase 9 (CAIX, MRQ-54), Claudin4 (CLDN4, 3E2C1), SMARCA2 (D9EB8), and SMARCA4 (EPNCIR111A).

All staining runs included appropriate internal positive controls and negative controls. Immunohistochemical expression was evaluated semi-quantitatively based on the proportion of positively stained tumor cells, using the following scale: negative (<1%), 1+ (1–25%), 2+ (26–75%), and 3+ (>75%).

### 2.4. Molecular NGS Study and Pathway-Based Clustering Analysis

Targeted next-generation sequencing (NGS) was performed on 17 ccRCC-R tumor samples using the Oncomine™ Comprehensive Assay V3 (Thermo Fisher Scientific, Waltham, MA, USA), which can detect 161 key cancer driver genes with enhanced kinase domain coverage and inclusion of genes involved in DNA damage repair. NGS was conducted at the Diagnostic Molecular Laboratory, Department of Pathology, Boston Medical Center, utilizing the Genexus Integrated Sequencer (Thermo Fisher Scientific, Waltham, MA, USA). Variant calling was performed using Thermo Fisher’s Ion Reporter software (Version 6.6.2.1).

Rhabdoid component-enriched regions were manually macro-dissected from formalin-fixed paraffin-embedded (FFPE) tissue, guided by hematoxylin and eosin (H&E)-stained reference slides, ensuring a minimum of 20% rhabdoid tumor cell content for molecular analysis.

Analytical sensitivity thresholds were defined as follows: a minimum variant allele frequency (VAF) of 5% for single-nucleotide variants (SNVs) and small insertions/deletions (indels), as well as a copy number of 6 or greater, for the detection of copy number alterations (amplifications). Detected variants were categorized into functional pathways relevant to ccRCC pathogenesis, including chromatin remodeling, DNA damage repair (DDR), PI3K/AKT/mTOR, CDK regulation, MAPK, TSC/NF, RB/p53/MYC, and NOTCH signaling. To assess functional pathway activation, a semi-quantitative burden score was assigned to each pathway in each tumor, based on the highest VAF among mutations in that pathway: score 0 (VAF < 5%), score 1 (5–20%), score 2 (21–50%), and score 3 (>50%). A heatmap was generated using seaborn.clustermap in Python (Version 3.8) to visualize pathway scores across tumors.

## 3. Results

### 3.1. Clinicopathological Features

Seventeen tumors of clear cell renal cell carcinoma with rhabdoid features (ccRCC-R) were included in this study. The cohort consisted of 11 male and 6 female patients, with a median age of 64 years (range: 42–86 years). The median tumor size was 8.5 cm (range: 6.0–16.5 cm), with 11 tumors located in the right kidney and 6 in the left. Twelve tumors were staged as pT3a, three as pT3b, and two as pT4, based on the AJCC 8th Edition criteria. Follow-up data were available for 12 patients, with a median follow-up duration of 37 months (range: 1–130 months). Ten patients developed distant metastases, including two patients who presented with synchronous distant metastases at diagnosis and eight who developed metastases during follow-up with histopathologic confirmation, with a median interval of 14 months (range: 3–69 months). The most common metastatic sites were the lungs (n = 7), followed by the liver (n = 3), bone (n = 3), lymph nodes (n = 2), contralateral kidney (n = 1), and adrenal gland (n = 1). Of the 12 patients with clinical follow-up, 9 were alive and 3 had died; however, no disease-related deaths were observed during the follow-up period. Three patients received immune checkpoint blockade (ICB) adjuvant therapy, and seven received systemic therapy, including VEGF inhibitor (n = 3), immunotherapy/ICB (n = 5), tyrosine kinase inhibitors (n = 2), and radiation therapy (n = 1). A summary of the clinicopathologic characteristics is provided in Table 1.

### 3.2. Histomorphologic Features and Immunohistochemical Results

A histopathologic evaluation of 17 ccRCC-R tumors demonstrated marked architectural and cytologic heterogeneity. All tumors were classified as WHO/ISUP grade 4, with high-grade components comprising 5% to 75% of the tumor volume. Detailed quantification of morphologic parameters, including the extent of low-grade classic ccRCC components, rhabdoid and sarcomatoid differentiation, other pleomorphic cells, and tumor necrosis, is summarized in Table 2. Rhabdoid features were identified in all tumors (100%), ranging from 5% to 70% of the tumor area. Morphologically, these features were defined by large, discohesive, and epithelioid cells with abundant eosinophilic cytoplasm, paranuclear intracytoplasmic inclusion, eccentric vesicular nuclei, and prominent nucleoli. Sarcomatoid differentiation was observed in five tumors, predominantly involving approximately 5% of the tumor volume, with one case showing up to approximately 20% involvement. Multinucleated giant cells were present in thirteen tumors (76%), frequently associated with increased nuclear pleomorphism. Tumor necrosis was identified in all tumors (100%), involving 5% to 40% of the tumor area.

**Figure 1:**
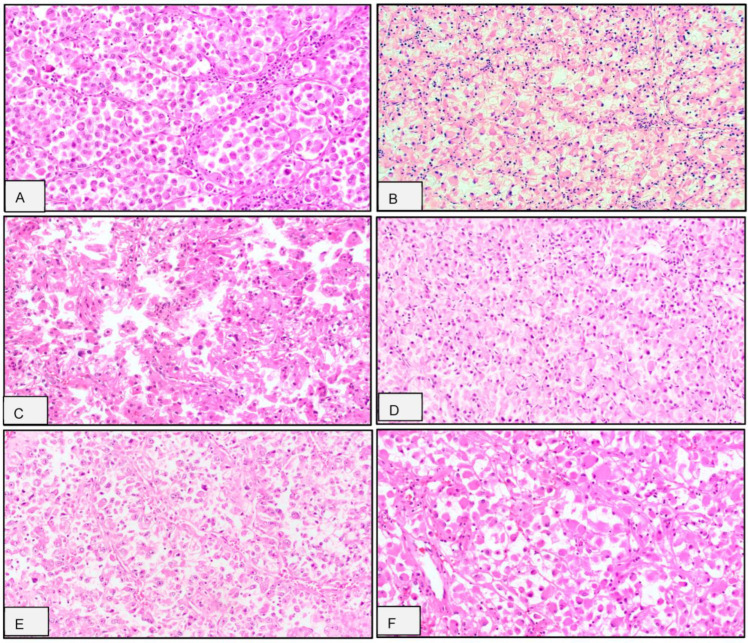
Histomorphologic features highlighting the architectural heterogeneity and rhabdoid cytology of clear cell renal cell carcinoma with rhabdoid features (ccRCC-R). (**A**) Alveolar growth pattern, ×200; (**B**) Trabecular growth pattern, ×100; (**C**) Pseudopapillary and translocation RCC-like growth pattern, ×200; (**D**) Solid/sheet-like growth pattern, ×200; and (**E**,**F**) Pseudopapillary growth pattern, ×100, ×200.

Architectural patterns associated with rhabdoid morphology (Figure 1 and Table 2) included predominantly solid/sheet-like growth in seven tumors (41%), alveolar/trabecular architecture in six tumors (35%), and pseudopapillary architecture in four tumors (24%).

**Figure 2:**
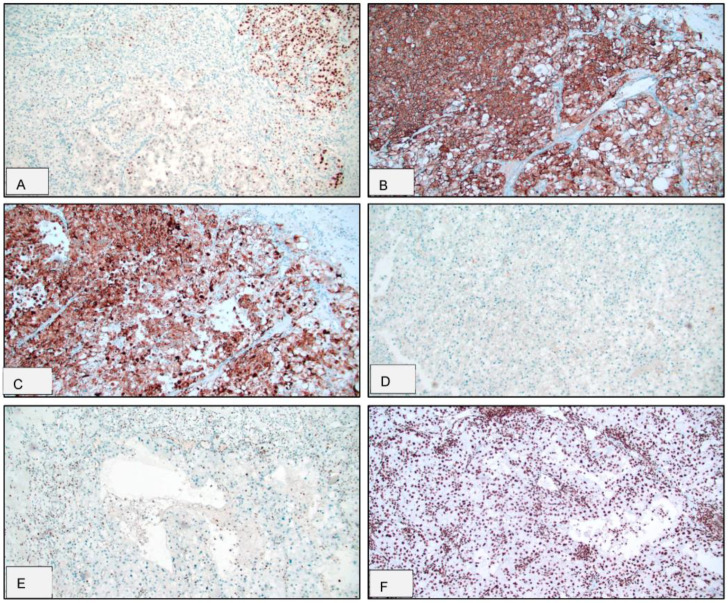
Immunoprofile of clear cell renal cell carcinoma with rhabdoid features (ccRCC-R). (**A**) Predominant loss of PAX8 expression, ×100; (**B**) Diffuse and complete membranous staining pattern for CAIX, ×100; (**C**) Retained cytoplasmic expression of cytokeratin AE1/AE3, ×100; (**D**) Loss of Claudin4 expression, ×100; (**E**) Loss of nuclear SMARCA2 staining, ×100; and (**F**) Retained nuclear expression of SMARCA4, ×100.

Immunohistochemical analysis was performed on tumor sections enriched for the rhabdoid component in all 17 ccRCC-R tumors (Figure 2). CAIX expression was strong (3+) in 13 tumors (76%), with reduced expression (2+ or 1+) observed in 4 tumors. SMARCA2 loss was identified in 13 tumors (76%), with weak (1+) retained expression in 2 tumors. SMARCA4 was strongly retained (3+) in all evaluable tumors. Claudin4 expression was absent in 11 tumors (65%) and was weak to moderate (1+ to 2+) in 5 tumors. AE1/AE3 was positive in 15 tumors (88%), with staining intensity ranging from 2+ to 3+. PAX8 expression was present in nine tumors (53%) but absent in seven tumors. As summarized in Table 3, the immunoprofiling of ccRCC-R reflects its high-grade dedifferentiated nature, characterized by frequent loss of Claudin4, SMARCA2, and PAX8, with the retention of AE1/AE3 and CAIX.

### 3.3. Molecular NGS Analysis and Pathway-Based Subclassification

All 17 tumors of ccRCC-R underwent targeted next-generation sequencing (NGS) performed specifically on tumor regions enriched for rhabdoid morphology (rROI). Rhabdoid cell elements demonstrated marked genomic heterogeneity, with all tumors displaying complex mutational profiles across multiple oncogenic pathways.

Chromatin remodeling gene mutations were identified in 9 of 17 tumors (53%), involving BAP1 mutations in 6 tumors (35%), SETD2 mutations in 4 tumors (24%), and an ARID1A mutation in 1 tumor (6%). DNA damage repair (DDR) pathway alterations were detected in six tumors (35%), including mutations in CDK12 (four tumors, 24%), PMS2 (three tumors, 18%), and MSH6 (two tumors, 12%). PI3K/AKT/mTOR pathway aberrations were present in 10 tumors (59%), predominantly involving FGFR4 mutations (8 tumors, 47%), MTOR mutations (2 tumors, 12%), and PTEN mutations (2 tumors, 12%). CDK pathway alterations, affecting CDK12 and CDKN2B-AS1, were observed in five tumors (29%). Mutations in the TSC/NF pathway, including TSC1, TSC2, NF1, and NF2, were identified in five tumors (29%). MAPK pathway activation through MAP2K2 truncating mutations was observed in five tumors (29%). RB/p53 pathway alterations were detected in one tumor (6%), and MYC amplification was seen in two tumors (12%). These findings highlight the molecular complexity of ccRCC-R, with frequent alterations in chromatin remodeling genes and critical oncogenic pathways. A detailed case-by-case summary is provided in Table 4.

**Figure 3:**
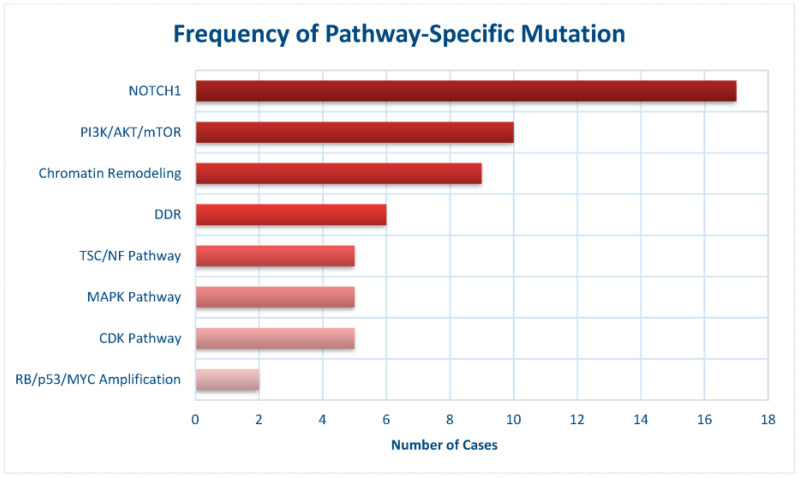
Frequency of pathway-based mutations in 17 cases of ccRCC-R.

Pathway-specific mutation frequencies are depicted in Figure 3. NOTCH1 alterations were the most frequent, followed by alterations in PI3K/AKT/mTOR pathway genes (59%), chromatin remodeling genes (53%), and DDR-related genes (35%). Additional alterations involved CDK pathway regulators (29%), MAPK pathway components (29%), and TSC/NF pathway genes (29%), as well as RB/p53 pathway genes and MYC amplifications (12%).

**Figure 4:**
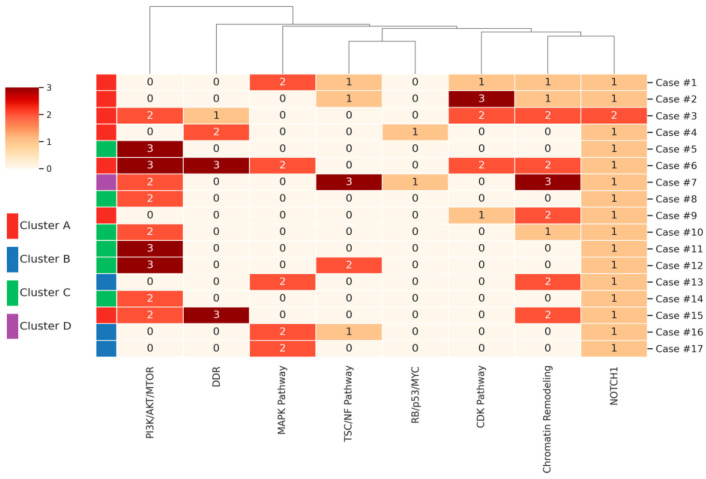
Unsupervised hierarchical clustering heatmap of ccRCC-R highlighting allele frequency–weighted pathway mutation burden and four molecular subgroups. This hierarchical heatmap illustrates allele frequency-weighted pathway mutation burdens (scale 0–3, reflected by color intensity) across individual tumors. Unsupervised clustering stratifies the 17 ccRCC-R tumors into four biologically distinct subgroups: Cluster A (DDR/Chromatin remodeling/CDK), Cluster B (MAPK-driven), Cluster C (mTOR/FGFR4-dominant), and Cluster D (RB/p53/MYC). Row color sidebars indicate cluster membership—red, blue, green, and purple, respectively—and reflect potential therapeutic implications.

Unsupervised hierarchical clustering based on allele frequency-weighted pathway mutation burden revealed four distinct molecular subgroups (Figure 4 and Table S1). A burden score matrix (0–3 per pathway) visualized mutation intensity across eight canonical oncogenic pathways. Cluster A, representing a multifocal high-burden subtype, included Tumors #1, #2, #3, #4, #6, #9, and #15. This group was defined by co-occurring alterations in chromatin remodeling, CDK, and DNA damage repair (DDR) pathways. Tumors in Cluster A frequently harbored mutations in genes such as BAP1, SETD2, ARID1A, CDK12, MSH6, and PMS2, often presenting in combination within individual tumors. Cluster B, the MAPK-driven subtype, encompassed Tumors #13, #16, and #17, with dominant MAPK pathway activation—most notably MAP2K2 truncating mutations—and a lower burden across other oncogenic axes. Cluster C, designated as the mTOR/PI3K-dominant subtype, comprised Tumors #5, #8, #10, #11, #12, and #14. These tumors were enriched for FGFR4, PTEN, and MTOR mutations, consistent with activated PI3K/AKT/mTOR signaling and the limited engagement of other pathways. Cluster D, a complex and genomically unstable subtype, included only Tumor #7, which exhibited high-level alterations across TSC1, TSC2, RB1, TP53, and MYC, indicative of a dedifferentiated and aggressive molecular phenotype. These distinct pathway-defined molecular classes highlight the heterogeneity of clear cell renal cell carcinoma with rhabdoid features (ccRCC-R) and suggest biologically and therapeutically meaningful subgrouping.

## 4. Discussion

Clear cell renal cell carcinoma with rhabdoid features (ccRCC-R) is increasingly recognized as a distinct and highly aggressive variant of ccRCC, characterized by profound histologic dedifferentiation, extensive intratumoral heterogeneity, and poor clinical outcomes [1,2,3]. Our study provides an integrative histomorphologic, immunophenotypic, and molecular analysis, offering new insights into the biological underpinnings of this aggressive phenotype.

Clinically, the majority of ccRCC-R tumors in our cohort presented at advanced pathologic stages (pT3–pT4) and demonstrated rapid metastatic progression (a median interval of 14 months), consistent with previous reports demonstrating that the presence of rhabdoid features markedly increases the risk of extrarenal extension and systemic dissemination [2]. These findings reinforce prior studies indicating that rhabdoid differentiation, even when focal, independently predicts decreased cancer-specific survival in ccRCC patients [13,14].

Histologically, ccRCC-R is characterized by the effacement of the classic nested architecture, with replacement by solid, sheet-like, or alveolar/trabecular growth patterns. These are often accompanied by the loss of the delicate vascular networks typical of conventional clear cell RCC. Pseudopapillary structures and geographic necrosis are also frequent, reflecting underlying tumor hypoxia and architectural dedifferentiation. Cytologically, the rhabdoid tumor cells are large, discohesive, and epithelioid, exhibiting abundant eosinophilic cytoplasm, paranuclear intracytoplasmic inclusion, eccentric vesicular nuclei, and prominent nucleoli.

High-grade cytologic features, in addition to rhabdoid differentiation, such as sarcomatoid transformation, large pleomorphic cells, and multinucleated giant cells, are frequently observed in ccRCC-R and often coexist within the same tumor [11]. This constellation of features highlights the profound morphologic heterogeneity of ccRCC-R and its progression toward highly aggressive phenotypes. Notably, the overlapping histologic features shared with other high-grade renal and mesenchymal neoplasms, such as MiTF family translocation RCC, fumarate hydratase-deficient RCC, epithelioid PEComa, and alveolar soft part sarcoma, can pose a significant diagnostic challenge and require thorough ancillary testing.

Immunophenotypic profiling supports this morphologic complexity. Rhabdoid tumor cells frequently exhibit partial loss of renal epithelial markers such as PAX8 and Claudin4 while generally retaining the expression of cytokeratin AE1/AE3 and the hypoxia-associated marker CAIX. Furthermore, loss of SMARCA2 expression with retained SMARCA4 was observed in the majority of cases. These findings are consistent with dedifferentiation-associated epigenetic dysregulation previously described in aggressive renal neoplasms [15,16]. The characteristic immunohistochemical profile reinforces the dedifferentiated nature of ccRCC-R and offers potential diagnostic utility in distinguishing it from histologic mimics.

Building on the clinicopathologic characterization of clear cell renal cell carcinoma with rhabdoid differentiation (ccRCC-R), our molecular analysis highlights the genomic complexity that likely contributes to its aggressive clinical behavior. In conventional ccRCC, the loss of chromosome 3p is a common early event, frequently leading to the simultaneous inactivation of key tumor suppressor genes, including PBRM1, BAP1, and SETD2, all of which are involved in chromatin remodeling and histone regulation. Among these, PBRM1 and SETD2 mutations often co-occur and are typically associated with a more indolent disease course. In contrast, BAP1 mutations are usually mutually exclusive with PBRM1 alterations and correlate strongly with high-grade morphology, aggressive behavior, and a higher risk of early metastasis [7,8]. Additional driver mutations involving histone-modifying enzymes (e.g., *KDM5C* and *KDM6A*), components of the PI3K/AKT/mTOR pathway (*TSC1*, *TSC2*, *MTOR*, *PIK3CA*, and *PTEN*), and TP53 further contribute to disease progression and reflect the molecular heterogeneity of ccRCC [17,18,19]. Tumors harboring BAP1 mutations or multiple co-occurring genomic alterations often exhibit increased metastatic potential, consistent with a biologically aggressive phenotype and a higher likelihood of high-grade transformation [18,20].

In our study cohort, targeted next-generation sequencing (NGS) of rhabdoid-enriched tumor regions revealed notable genetic heterogeneity, with recurrent alterations involving multiple oncogenic pathways. Consistent with prior studies highlighting the potential role of chromatin dysregulation in aggressive renal neoplasms, we frequently identified mutations in chromatin remodeling genes, particularly BAP1 and SETD2, both of which have often been associated with rhabdoid features in ccRCC [8,21,22]. Additionally, we noted a recurrent co-occurrence of alterations in the DNA damage repair (DDR) pathway, including *CDK12*, *PMS2*, and *MSH6*. These findings suggest that impaired genomic maintenance contributes to the underlying biology of ccRCC-R [23].

In addition to alterations involving chromatin remodeling and DNA damage repair (DDR) pathways, our data also suggest a role for signaling pathway dysregulation in the biology of ccRCC-R. Recurrent mutations in *FGFR4*, *PTEN*, and *MTOR*, as well as truncating alterations in *MAP2K2*, point to the frequent involvement of the PI3K/AKT/mTOR and MAPK signaling cascades. These findings are consistent with the hypothesis that multiple oncogenic pathways may contribute to both tumor progression and therapeutic resistance in ccRCC-R. Collectively, our results underscore the potential utility of molecular stratification in guiding biomarker-driven targeted treatment strategies for this aggressive tumor subtype.

*NOTCH1* mutations were identified in all tumors within our ccRCC-R cohort, suggesting that NOTCH pathway dysregulation may contribute to rhabdoid dedifferentiation. Although the functional impact of these alterations in renal tumors remains unclear, their consistent presence in rhabdoid components warrants further investigation. Recent studies have begun to explore *NOTCH1* in ccRCC biology. Zhuang et al. reported that *NOTCH1* may promote tumorigenesis and progression via the AKT/mTOR pathway, with higher expression associated with advanced stage, higher grade, and larger tumor size [24]. Similarly, Zhang et al. identified NOTCH signaling-related lncRNAs implicated in ccRCC pathogenesis, with elevated expression linked to poor prognosis, immune checkpoint activation, and drug resistance [25]. While these findings are not specific to the rhabdoid subtype, they provide a basis for considering a role for *NOTCH1* in aggressive ccRCC variants. Further studies are needed to clarify its role in rhabdoid transformation and its potential as a therapeutic target.

To further elucidate the potential biological implications of these molecular alterations, unsupervised clustering analysis was performed, revealing distinct molecular subgroups within ccRCC-R. These clustering results underscore the molecular heterogeneity of ccRCC-R and may offer a framework for stratifying patients into biologically and therapeutically relevant subgroups. One such subgroup, Cluster A, was characterized by co-occurring alterations in chromatin remodeling and DNA damage repair gene alterations that may be associated with increased immunogenicity and potential sensitivity to therapies targeting synthetic lethality, such as PARP inhibitors or immune checkpoint blockade (ICB) [26]. In our cohort, seven patients exhibited this molecular profile; although most were either lost to follow-up or did not receive systemic therapy, patient #15, who was treated with immune checkpoint blockade (ICB), achieved no evidence of disease (NED) at three years post-treatment. While anecdotal, this observation raises the possibility of clinical benefit in selected cases. This finding aligns with emerging evidence showing robust responses to ICB in renal cell carcinoma with rhabdoid or sarcomatoid differentiation. Additionally, five cases in our cohort exhibited focal sarcomatoid dedifferentiation, although none were classified within Cluster A. Of these, two patients (Cases #16 and #17) received immune checkpoint blockade (ICB) and remained disease-free at 29 and 30 months of follow-up, respectively. While the CheckMate 214 trial [27] demonstrated that even minimal sarcomatoid components can predict ICB responsiveness, the small sample size and absence of targeted molecular profiling of sarcomatoid regions in our study limit direct comparisons between rhabdoid and sarcomatoid subtypes.

Supporting this notion, multiple independent cohorts have shown that both rhabdoid and sarcomatoid differentiation may serve as prognostic biomarkers for survival and immunotherapy response [6,14,28,29]. Although these findings are not specific to ccRCC-R, they highlight the potential benefit of ICB in tumors with dedifferentiated histology. Taken together, our observations support the integration of both molecular and histologic features in future studies to refine risk stratification and guide treatment selection in ccRCC-R.

In contrast, Cluster B tumors, which were enriched for MAPK pathway alterations such as *MAP2K2* mutations, may reflect a distinct pattern of oncogenic signaling. While ccRCC is not typically associated with canonical MAPK/ERK driver mutations, aberrant activation of this pathway has been implicated in resistance to both VEGF-targeted therapies and immune checkpoint inhibitors. Although MEK inhibitors have demonstrated limited efficacy as monotherapy in ccRCC, preclinical and early-phase clinical studies have begun to explore combination approaches, such as MEK inhibitors with VEGF inhibitors or immune checkpoint blockade, with modest results reported to date [30]. These preliminary findings suggest that further investigation into rational combination strategies, potentially tailored to molecular subtypes such as Cluster B, may be warranted in ccRCC-R.

The predominance of *FGFR4*, *PTEN*, and *MTOR* alterations in the mTOR/PI3K-dominant subgroup (Cluster C) suggests potential benefit from targeted kinase inhibition [31,32]. In our cohort, two patients (#10 and #14) received adjuvant tyrosine kinase inhibitor (TKI) therapy. Patient #10, despite experiencing a non-disease-related death (DNAD), demonstrated a favorable treatment response during the two years prior to death. Patient #14 remained NED at 52 months following combined ICB and TKI therapy. These observations suggest that selected patients within this molecular subgroup may derive clinical benefit from kinase-targeted or combination regimens.

While immune checkpoint inhibitors are generally considered more effective than TKIs in renal cell carcinomas with sarcomatoid or rhabdoid features, our findings raise the possibility that TKI-based therapy may retain therapeutic relevance in select cases, particularly in Cluster C tumors characterized by PI3K/mTOR pathway activation. Notably, Cluster C comprised 6 of 17 cases (35%) in our cohort, highlighting the potential value of molecular profiling in identifying patients who may benefit from kinase-directed therapies, either as monotherapy or in combination with ICB. These observations support the potential utility of a pathway-informed approach to treatment selection in ccRCC-R, guided by the dominant oncogenic alterations within each molecular subgroup.

Cluster D was characterized by a genomically unstable and aggressive phenotype, defined by concurrent alterations in *RB1*, *TP53*, *TSC1/TSC2*, and *MYC* amplification—changes previously associated with poor outcomes and therapeutic resistance in renal cell carcinoma [5,33]. These co-occurring alterations suggest the disruption of cell cycle control and oncogenic signaling, potentially limiting response to standard therapies. In our cohort, Case #7 exemplified this profile, with additional mutations in chromatin remodeling, DDR pathways, and the mTOR/PI3K axis. The patient developed liver metastases within three months of surgery and was lost to follow-up shortly after starting VEGF-targeted therapy. While treatment response could not be fully assessed, the clinical and molecular features point to a high-risk subset of ccRCC-R that may require alternative therapeutic strategies.

These findings highlight the potential value of integrated molecular profiling in the clinical management of ccRCC-R. The observed biological and genomic heterogeneity, ranging from immuno-responsive to treatment-resistant phenotypes, supports the need for a pathway-based stratification framework to guide therapy. For example, Cluster A may be more amenable to immune checkpoint blockade, Cluster C may benefit from kinase inhibitors, and Cluster D appears to represent a high-risk subgroup with limited response to conventional treatment. Together, these distinctions support the rationale for precision oncology approaches informed by molecular characterization. Prospective studies incorporating functional validation and clinical correlation will be important for translating these insights into effective personalized strategies for this aggressive tumor subtype.

While this study provides preliminary insights into the molecular landscape of ccRCC-R, several limitations should be acknowledged. First, the relatively small sample size reflects the rarity of rhabdoid differentiation in ccRCC and the complexity of assembling a well-annotated cohort specific to this aggressive histologic subtype. Second, although targeted next-generation sequencing yielded meaningful information on recurrent genomic alterations, the use of a focused gene panel limited the scope of detectable mutational events and structural variations, potentially underestimating the full extent of genomic complexity. Third, the relatively short follow-up duration and limited number of clinical endpoints constrained the strength of clinicopathologic correlations and precluded definitive prognostic conclusions. Further studies incorporating larger multi-institutional cohorts, more comprehensive genomic platforms, and functional validation will be essential to build upon these findings and to support the development of refined pathway-informed therapeutic strategies for patients with ccRCC-R.

## 5. Conclusions

In summary, rhabdoid differentiation in clear cell renal cell carcinoma (ccRCC-R) appears to define a morphologically and molecularly distinct high-risk phenotype characterized by notable genomic heterogeneity and activation of diverse oncogenic pathways. Our findings suggest that integrating histomorphologic assessment with targeted molecular profiling can provide meaningful insights into tumor biology, support pathway-based stratification, and help identify potential therapeutic opportunities. Certain subgroups, such as those with DNA damage repair deficiencies, may be more responsive to immune checkpoint blockade, while tumors with PI3K/mTOR alterations could benefit from kinase-directed therapies. Conversely, tumors exhibiting complex genomic instability and cell cycle dysregulation may be less responsive to current treatment modalities and could require alternative or investigational strategies. These observations highlight the potential role of precision oncology in the management of ccRCC-R. Further work will be needed to refine molecular subclassification and to develop biologically informed pathway-specific approaches aimed at improving outcomes related to this challenging disease.

## Figures and Tables

**Table 1:** Summary of clinicopathological and immunohistochemical features of clear cell renal cell carcinoma with rhabdoid features (ccRCC-R).

Case (N)	Sex (M/F)	Age (yrs)	Laterality (L/R))	Tumor Size (cm)	WHO/ ISUP Grade	Pathologic Stage (pTNM)			Adjuvant Therapy	Systemic Therapy	Follow-Up (Months)	Clinical Outcome
1	M	74	L	8.5	4	pT3a	Nx	Mx	No	No	1	LTFU
2	F	62	R	6.5	4	pT3a	Nx	Mx	No	No	38	AWD
3	F	86	R	6.4	4	pT4	N0	Mx	No	No	94	DNAD
4	M	42	R	7	4	pT3b	N1	Mx	No	No	1	LTFU
5	F	71	R	6	4	pT3a	N1	Mx/M1(6 mons)	No	No	6	LTFU
6	F	66	R	9.5	4	pT3a	Nx	Mx/M1(49 mons)	No	No	53	DNAD
7	M	54	R	15	4	pT3b	Nx	Mx/M1(3 mons)	No	VEGFi (Pazo)	4	LTFU
8	M	52	L	5	4	pT3a	Nx	Mx/M1(14 mons)	No	VEGFi (Pazo); Radiation; ICB (Nivo + Ipi)	130	AWD
9	M	49	R	7	4	pT3a	Nx	Mx/M1(69 mons)	No	No	115	AWD
10	M	72	R	9	4	pT3b	N0	M1(lung)	No	VEGFi (Pazo); ICB (Nivo + Ipi); TKI (Cabo)	28	DNAD
11	M	63	L	10.5	4	pT3a	Nx	Mx	No	No	55	NED
12	M	46	L	10	4	pT3a	N0	Mx/M1(5 mons)	No	ICB (Nivo + Ipi)	5	LTFU
13	M	66	R	11	4	pT3a	N1	Mx/M1(12 mons)	No	ICB (Nivo + Ipi)	54	NED
14	F	58	R	11	4	pT4	N0	Mx/M1(18 mons)	ICB (Nivo + Ipi)	TKI (Lenv); mTORi (Everol)	52	AWD
15	F	66	L	8.3	4	pT3a	N0	M1(adrenal)	NA	ICB (Nivo)	37	NED
16	M	60	R	7.6	4	pT3a	Nx	Mx	ICB (Pembro)	NA	30	NED
17	M	65	L	16.5	4	pT3a	Nx	Mx	ICB (Pembro)	NA	29	NED

Note: M, male; F, female; R, right; L, left; Nx, regional lymph nodes cannot be assessed; Mx, distant metastasis cannot be assessed; Mx/M1: patients developed metastases during postoperative follow-up; VEGFi (Pazo), Vascular Endothelial Growth Factor inhibitor (Pazopanib); ICB (Nivo + Ipi), immune checkpoint blockade (Nivolumab + Ipilimumab); TKI (Lenv), tyrosine kinase inhibitor (Lenvatinib); mTORi (Everol), mammalian target of rapamycin inhibitor (Everolimus); LTFU, lost to follow-up; NED, no evidence of disease; AWD, alive with disease; DNAD, death not associated with disease.

**Table 2:** WHO/ISUP grade and detailed histomorphologic characterization of 17 cases of clear cell renal cell carcinoma with rhabdoid features (ccRCC-R).

Case (N)	WHO/ ISUP Grade 1/2 (%)	WHO/ ISUP Grade 3 (%)	WHO/ISUP Grade 4						Tumor Necrosis (%)
			Rhabdoid Features		Sarcomatoid Features (%)	Pleomorphic Cells *	Giant Cells (pleo/mono)	Total (%)	
			%	Architectures					
1	10	60	10	Solid/sheet-like	Np	5	Gp	15	15
2	20	10	25	Alveolar/trabecular	Np	<5	Gp/Gm	30	40
3	5	15	30	Solid/sheet-like	Np	<5	Gp	40	40
4	20	30	25	Solid/sheet-like	Np	<5	Gp	30	20
5	20	50	10	Pseudopapillary	<5	<5	Gp	20	10
6	30	30	15	Solid/sheet-like	Np	Np	Gm	20	20
7	10	50	15	Solid/sheet-like	<5	<5	Gm	20	20
8	60	10	20	Solid/sheet-like	Np	Np	Np	20	10
9	20	30	30	Alveolar	Np	<10	Np	40	10
10	40	50	5	Solid/sheet-like	Np	Np	Np	5	5
11	10	30	20	Pseudopapillary	20	<5	Gp	40	20
12	5	15	60	Alveolar/trabecular	Np	<10	Gp	70	10
13	20	35	30	Alveolar/trabecular	Np	<5	Np	35	10
14	5	15	70	Pseudopapillary	Np	<5	Gp	75	5
15	10	20	40	Pseudopapillary	Np	Np	Gp	40	30
16	30	35	10	Alveolar	<5	<10	Gp	25	10
17	5	30	20	Alveolar	<5	<5	Gp	25	40

Note: Pleomorphic cells *, pleomorphic tumor cells without rhabdoid or sarcomatoid morphology; Gp, giant pleomorphic cells; Gm, giant monomorphic cells; Np, not present.

**Table 3:** Summary of immunohistochemical features of rhabdoid components in 17 cases of clear cell renal cell carcinoma (ccRCC-R).

Case (N)	PAX8	CAIX	AE1/AE3	Claudin4	SMARCA2	SMARCA4
1	−	3+	3+	−	−	3+
2	−	3+	3+	2+	−	3+
3	−	3+	−	−	−	3+
4	−	2+	3+	−	−	3+
5	2+	1+	2+	−	1+	3+
6	2+	3+	3+	−	1+	3+
7	−	2+	2+	−	−	3+
8	−	3+	3+	2+	−	3+
9	2+	3+	3+	2+	3+	3+
10	3+	3+	3+	2+	−	3+
11	−	3+	3+	−	−	3+
12	NP	3+	3+	−	−	3+
13	2+	3+	3+	−	−	3+
14	3+	3+	3+	−	−	3+
15	2+	3+	NP	NP	NP	NP
16	2+	3+	3+	1+	−	3+
17	2+	1+	3+	−	−	3+

Note: NP, not preformed; − (negative, <1% positive cells); 1+ (1–25% positive cells); 2+ (26–75% positive cells); and 3+ (>75% positive cells).

**Table 4:** Molecular alterations by pathway: case-by-case targeted NGS analysis.

Tumor (N)	Chromatin Remodeling	DDR	PI3K/AKT/mTOR	CDK Pathway	TSC/NF Pathway	RB/p53	MYC Amplification	MAPK Pathway	NOTCH1 Pathway
1	*BAP1*	*CDK12*	--	*CDK12*	*NF2*	--	--	*MAP2K2*	*NOTCH1*
2	*SETD2*	*CDK12*	--	*CDK12*	*NF1*	--	--	--	*NOTCH1*
3	*BAP1*	*PMS2/* *CDK12*	*PTEN*	*CDK12/CDKN2B-AS1*	--	--	--	--	*NOTCH1*
4	--	*MHS6*	--	--	--	--	*MYC-CN*	--	*NOTCH1*
5	--	--	*FGFR4/* *MTOR*	--	--	--	--	--	*NOTCH1*
6	*BAP1/* *ARID1A*	*PMS2/* *CDK12*	*FGFR4*	*CDK12/CDKN2B-AS1*	--	--	--	*MAP2K2*	*NOTCH1*
7	*BAP1*	--	*FGFR4*	--	*TSC1/TSC2*	*RB1/p53*	*MYC-CN*	--	*NOTCH1*
8	--	--	*PTEN*	--	--	--	--	--	*NOTCH1*
9	*SETD2*	--	--	*CDKN2B-AS1*	--	--	--	--	*NOTCH1*
10	*SETD2*	--	*FGFR4/* *PTEN*	--	--	--	--	--	*NOTCH1*
11	--	*-*	*FGFR4*	--	--	--	--	--	*NOTCH1*
12	--	--	*FGFR4*	--	*TSC1*	--	--	--	*NOTCH1*
13	*BAP1*	--		--	--	--	--	*MAP2K2*	*NOTCH1*
14	--	--	*FGFR4/* *MTOR*	--	--	--	--	--	*NOTCH1*
15	*BAP1/* *SETD2*	*PMS2/* *MHS6*	*FGFR4*	--	--	--	--	--	*NOTCH1*
16	--	--	--	--	*TSC1*	--	--	*MAP2K2*	*NOTCH1*
17	--	--	--	--	--	--	--	*MAP2K2*	*NOTCH1*

Note: The font color for *CDK12* indicated its role in both DNA damage response (DDR) and cyclin-dependent kinase (CDK) pathways.

## Data Availability

The original contributions presented in this study are included in the article/Supplementary Material. Further inquiries can be directed to the corresponding author.

## Supplementary Materials

The following supporting information is available at: https://www.mdpi.com/article/10.3390/cancers17172744/s1, Table S1: Targeted NGS analysis data of 17 ccRCC-R cases.

## Abbreviations

The following abbreviations are used in this manuscript:

ccRCC	Clear cell renal cell carcinoma
ccRCC-R	Clear cell renal cell carcinoma with rhabdoid features
FFPE	Formalin-fixed paraffin-embedded
NGS	Next-generation sequencing
WHO/ISUP	World Health Organization/International Society of Urological Pathology
VAF	Variant allele frequency
SNV	Single-nucleotide variants
DDR	DNA damage repair
VEGFi (Pazo)	VEGF inhibitor (Pazopanib)
ICB (Nivo + Ipi)	Immune checkpoint blockade (Nivolumab + Ipilimumab)
TKI (Cabo)	Tyrosine kinase inhibitor (Cabozantinib)
LTFU	Lost to follow-up
NED	No evidence of disease
AWD	Alive with disease
DNAD	Death not associated with disease
